# Omega-3 fatty acid prevents the development of heart failure by changing fatty acid composition in the heart

**DOI:** 10.1038/s41598-020-72686-0

**Published:** 2020-09-23

**Authors:** Haruhiro Toko, Hiroyuki Morita, Masanori Katakura, Michio Hashimoto, Toshiyuki Ko, Satoshi Bujo, Yusuke Adachi, Kazutaka Ueda, Haruka Murakami, Masato Ishizuka, Jiaxi Guo, Chunxia Zhao, Takayuki Fujiwara, Hironori Hara, Norifumi Takeda, Eiki Takimoto, Osamu Shido, Mutsuo Harada, Issei Komuro

**Affiliations:** 1 Department of Cardiovascular Medicine, Graduate School of Medicine, The University of Tokyo, 7-3-1 Hongo, Bunkyo-ku, Tokyo 113-8655 Japan; 2 Department of Advanced Translational Research and Medicine in Management of Pulmonary Hypertension, Graduate School of Medicine, The University of Tokyo, 7-3-1 Hongo, Bunkyo-ku, Tokyo 113-8655 Japan; 3 Department of Environmental Physiology, Faculty of Medicine, Shimane University, 89-1 Enyacho, Izumo, Shimane 693-8501 Japan; 4 Laboratory of Nutritional Physiology, Department of Pharmaceutical Sciences, Faculty of Pharmacy and Pharmaceutical Sciences, Josai University, 1-1 Keyakidai, Sakado, Saitama 350-0295 Japan; 5 Department of Advanced Clinical Science and Therapeutics, Graduate School of Medicine, The University of Tokyo, 7-3-1 Hongo, Bunkyo-ku, Tokyo 113-8655 Japan

**Keywords:** Cardiology, Molecular medicine

## Abstract

Some clinical trials showed that omega-3 fatty acid (FA) reduced cardiovascular events, but it remains unknown whether omega-3 FA supplementation changes the composition of FAs and their metabolites in the heart and how the changes, if any, exert beneficial effects on cardiac structure and function. To clarify these issues, we supplied omega-3 FA to mice exposed to pressure overload, and examined cardiac structure and function by echocardiography and a proportion of FAs and their metabolites by gas chromatography and liquid chromatography-tandem mass spectrometry, respectively. Pressure overload induced cardiac hypertrophy and dysfunction, and reduced concentration of all FAs’ components and increased free form arachidonic acid and its metabolites, precursors of pro-inflammatory mediators in the heart. Omega-3 FA supplementation increased both total and free form of eicosapentaenoic acid, a precursor of pro-resolution mediators and reduced free form arachidonic acid in the heart. Omega-3 FA supplementation suppressed expressions of pro-inflammatory cytokines and the infiltration of inflammatory cells into the heart and ameliorated cardiac dysfunction and fibrosis. These results suggest that omega-3 FA-induced changes of FAs composition in the heart have beneficial effects on cardiac function via regulating inflammation.

## Introduction

Various heart diseases such as hypertensive heart disease, ischemic heart disease and valvular heart disease finally result in heart failure^1^. Despite extensive studies and drug development, heart failure is still a leading cause of death in the world. Many mechanisms of heart failure have been reported such as ischemia, abnormal calcium handling, cardiomyocyte death, increase of reactive oxygen species (ROS) and mitochondrial dysfunction^2–6^. Recently, inflammation has been reported to be critically involved in the development of cardiac hypertrophy and heart failure^4,5,7–9^. Serum concentrations of pro-inflammatory cytokines such as TNFα, IL-1β and IL-6 as well as biomarkers such as CRP and galectin-3 have been reported to be elevated in patients with heart failure^10,11^, and their increases were attenuated by some therapeutic agents for heart failure such as angiotensin converting enzyme inhibitors and β blockers^11^.

Fatty acids (FAs) have three major biological roles such as an energy source, cellular membrane components and lipid mediators^12^. Considering that the heart beats every second, FAs are important as a source of sufficient energy and as a component of cellular membranes. Among lipid mediators, omega-3 FA and omega-6 FA have attracted much attention as sources of lipid mediators of inflammation^7,13–17^. Under basal conditions, these FAs are mainly located at sn-2 position of phospholipids in a cellular membrane. Once inflammatory stresses are imposed on a tissue, activated phospholipase A2 (PLA2) hydrolyzes the sn-2 ester bond of membrane phospholipids, and releases free form FAs such as arachidonic acid (ARA) from omega-6 FAs, and eicosapentaenoic acid (EPA) and docosahexaenoic acid (DHA) from omega-3 FAs. These free forms of FAs are modified by cyclooxygenase (COX)-2, 5-lipoxygenase (LOX) and 12/15-LOX, and are eventually turned into biological active lipid mediators^14–18^. It has been reported that ARA-derived metabolites are pro-inflammation, while EPA- and DHA-derived ones are pro-resolution^13–17^. Some metabolites have been reported to play a critical role in the development of cardiac hypertrophy and heart failure by regulating inflammatory reaction^19–21^. Recently, clinical trials showed that omega-3 FA supplementation reduced cardiovascular events^22–26^, but the underlying molecular mechanisms remain unknown.

In this study, we examined whether omega-3 FA supplementation has any effects on the composition of FA in the heart and whether these changes have beneficial effects on cardiac function and remodeling.

## Results

### Omega-3 FA ameliorates cardiac dysfunction induced by pressure overload

**Figure 1:**
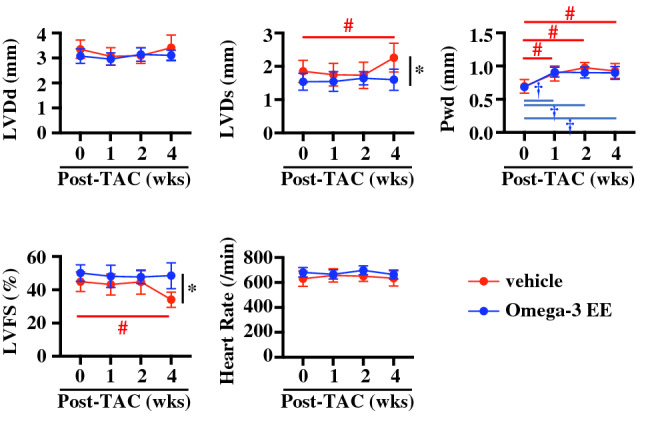
Omega-3 FA ameliorates cardiac dysfunction induced by pressure overload. Echocardiogram was performed to check cardiac morphology and function until 4 weeks (wks) after TAC operation. n = 6–12. *p < *0.05* compared between vehicle and Omega-3 EE groups. ^#^*p* < *0.05* compared with vehicle group before TAC*,* ^†^*p* < *0.05* compared with Omega-3 EE group before TAC. LVDd, left ventricular dimension at diastole. LVDs, left ventricular dimension at systole. Pwd, left ventricular posterior wall thickness at diastole. LVFS, left ventricular fractional shortening.

**Figure 2:**
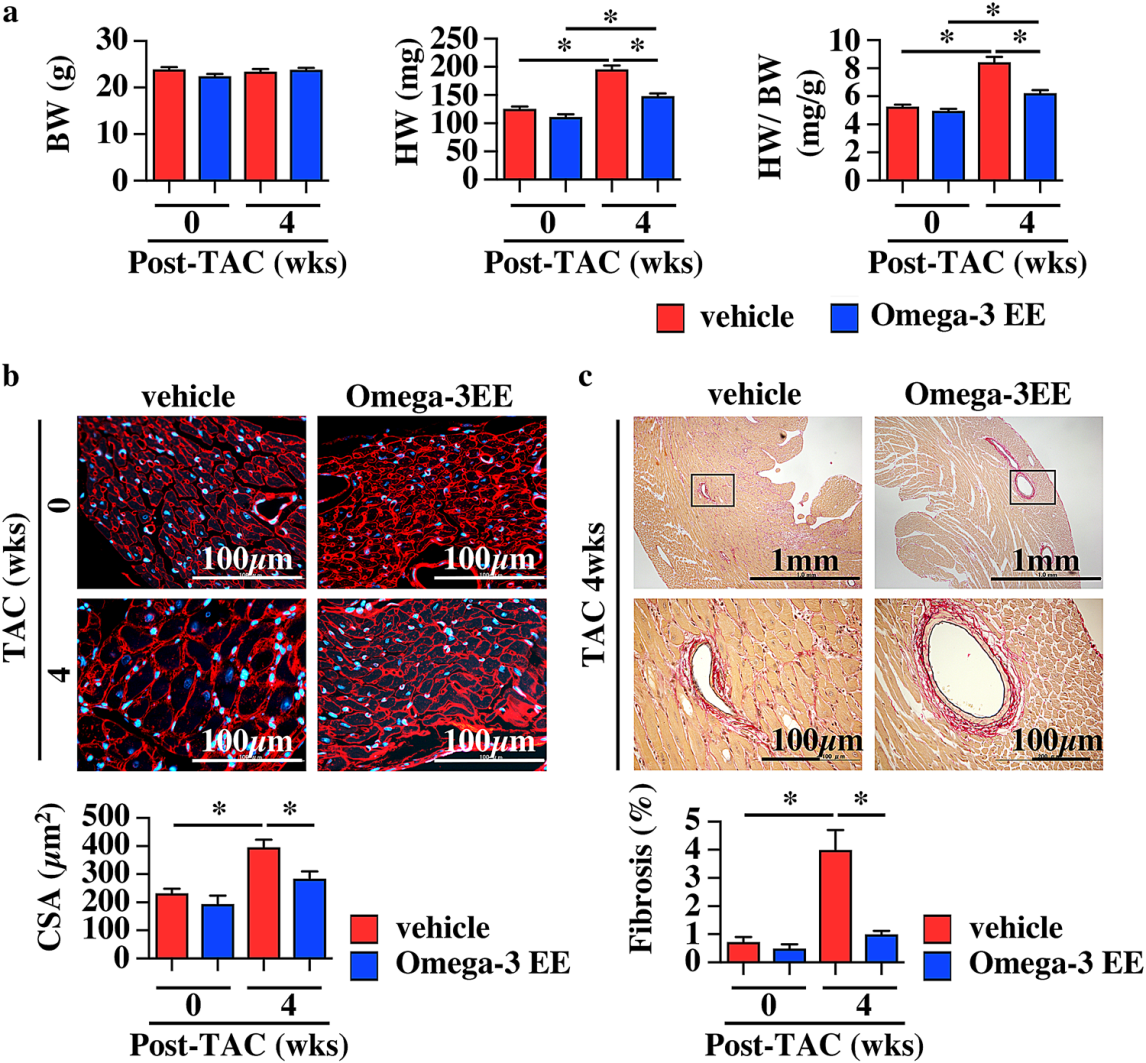
Omega-3 FA ameliorates cardiac hypertrophy induced by pressure overload. (**a**) Body weight (BW), heart weight (HW) and HW to BW ratio before and 4 weeks (wks) after TAC surgery with or without Omega-3 EE. n = 11–18. *p < *0.05*. (**b**) Cardiomyocyte surface area (CSA) was evaluated by wheat germ agglutinin stain. n = 5–12. * *p* < *0.05*. (**c**) Percent fibrosis in the heart at 4 weeks after operation was evaluated by Elastica van Gieson stain. n = 4–9. *p < *0.05*.

To assess the change of FA composition during a process of cardiac remodeling under pathological stresses, we firstly made the murine model of cardiac hypertrophy induced by constricting transverse aorta (TAC)^27^. We treated mice with vehicle and omega-3 FAs, major components of FAs in cardiomyocyte^20,28^ to clarify whether FA composition had some effects on cardiac function and structure. We administered Omega-3 acid ethyl esters (Omega-3 EE), a compound containing both EPA and DHA, 1.5 mg/g of body weight once a day. Although this dose is ~twice of a clinical dose in human^29^, this dose which had been reported to have a triglyceride-lowering effect in a rodent^30^ was adopted in this study. An echocardiogram showed that wall thickness of the left ventricle was hypertrophic with normal systolic function at 1 and 2 weeks after TAC and that left ventricular dimensions were enlarged at systole and cardiac systolic function was impaired at 4 weeks in vehicle-treated group (Fig. 1). On the other hand, Omega-3 EE inhibited the dilatation of cardiac dimension at systole and improved cardiac function at 4 weeks after operation (Fig. 1). Omega-3 EE also attenuated an increase in the heart weight (HW) to body weight (BW) ratio at 4 weeks after TAC (Fig. 2a). Tissue examinations revealed that Omega-3 EE suppressed pressure overload-induced cardiomyocyte hypertrophy and cardiac fibrosis (Fig. 2b,c). These results indicate that Omega-3 EE protects cardiac remodeling under pathological stresses.

### Omega-3 FA supplementation changes the proportion of FAs under pathological condition

**Figure 3:**
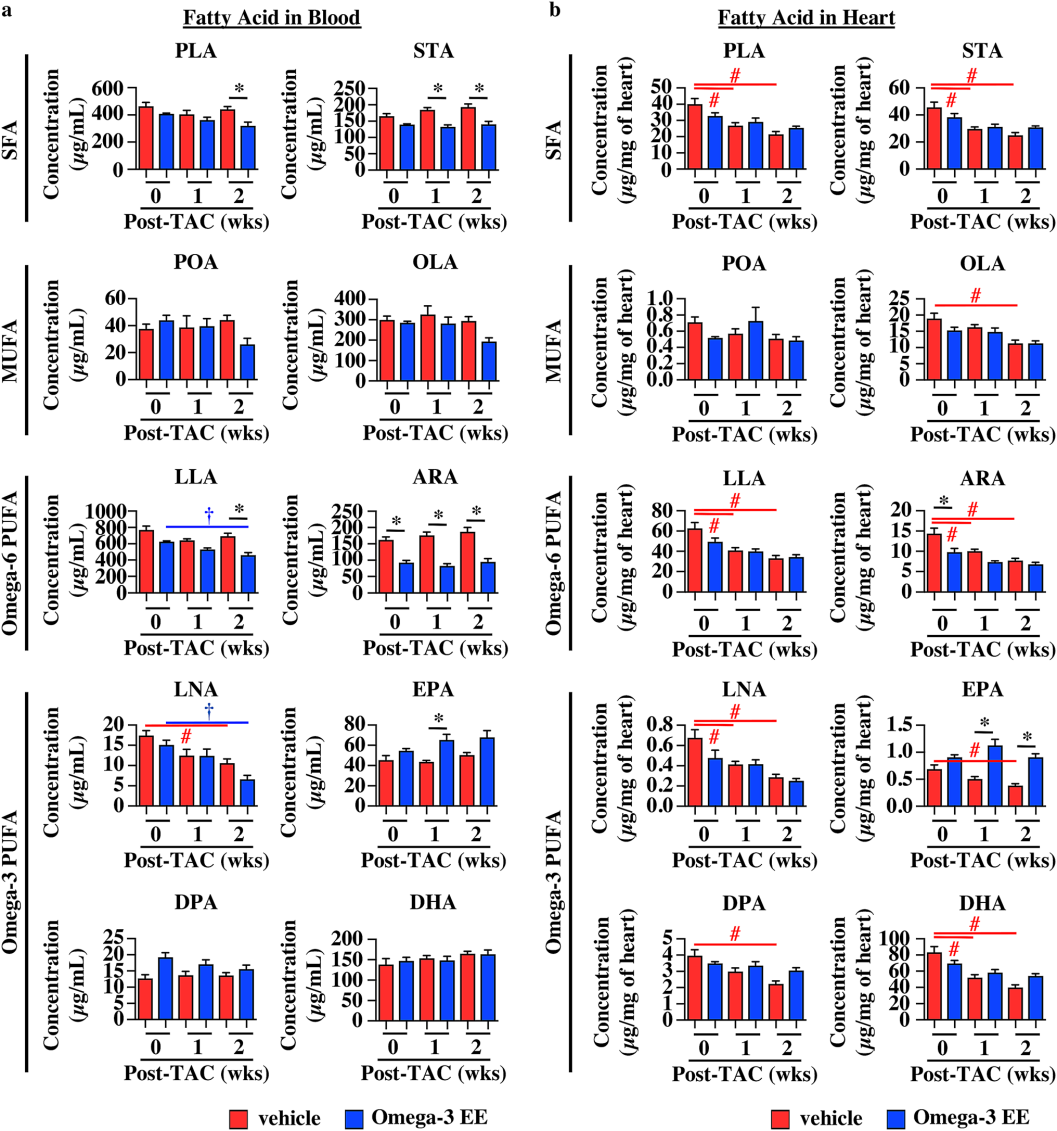
Pressure overload and Omega-3 FA supplementation change the proportion of FAs in blood and heart. (**a,b**) Gas chromatography revealed FA concentration in blood (**a**) and heart (**b**) before (0), and 1 and 2 weeks (wks) after TAC surgery. n = 4–6. * *p* < *0.05* compared between vehicle and Omega-3 EE groups. ^#^*p* < *0.05* compared with vehicle group before TAC*,* ^†^*p* < *0.05* compared with Omega-3 EE group before TAC. PLA, Palmitic acid. STA, Stearic acid. POA, Palmitoleic acid. OLA, Oleic acid. LLA, Linoleic acid. ARA, Arachidonic acid. LNA, Linolenic acid. EPA, Eicosapentaenoic acid. DPA, Docosapentaenoic acid. DHA, Docosahexaenoic acid. SFA, saturated fatty acid. MUFA, mono-unsaturated fatty acid. PUFA, poly-unsaturated fatty acid.

To know the mechanisms of how Omega-3 EE protects the heart from pressure overload, we first examined concentration of FAs before, and 1 and 2 weeks after TAC operation when there was no difference in left ventricular wall thickness and cardiac systolic function between vehicle and Omega-3 EE groups (Fig. 1). We used gas chromatography to evaluate compositional change of each FA in blood and heart^31,32^. Plasma levels of almost all FAs were not affected by pressure overload in vehicle group (Fig. 3a). On the other hand, all FAs were significantly decreased in the heart according to time after pressure overload in vehicle group (Fig. 3b).

In blood, there were less ARA and more EPA in Omega-3 EE group as compared with vehicle group (Fig. 3a). In the heart, concentrations of both ARA and DHA were not different between vehicle and Omega-3 EE groups under stress condition, while concentrations of EPA were higher in Omega-3 EE group than those in vehicle group (Fig. 3b). These results indicate that oral supplementation of Omega-3 EE changes the proportion of FAs and in particular increases EPA concentration in the pressure overloaded heart.

### Omega-3 FA treatment augments the increase in a free form EPA in the heart exposed with high blood pressure

**Figure 4:**
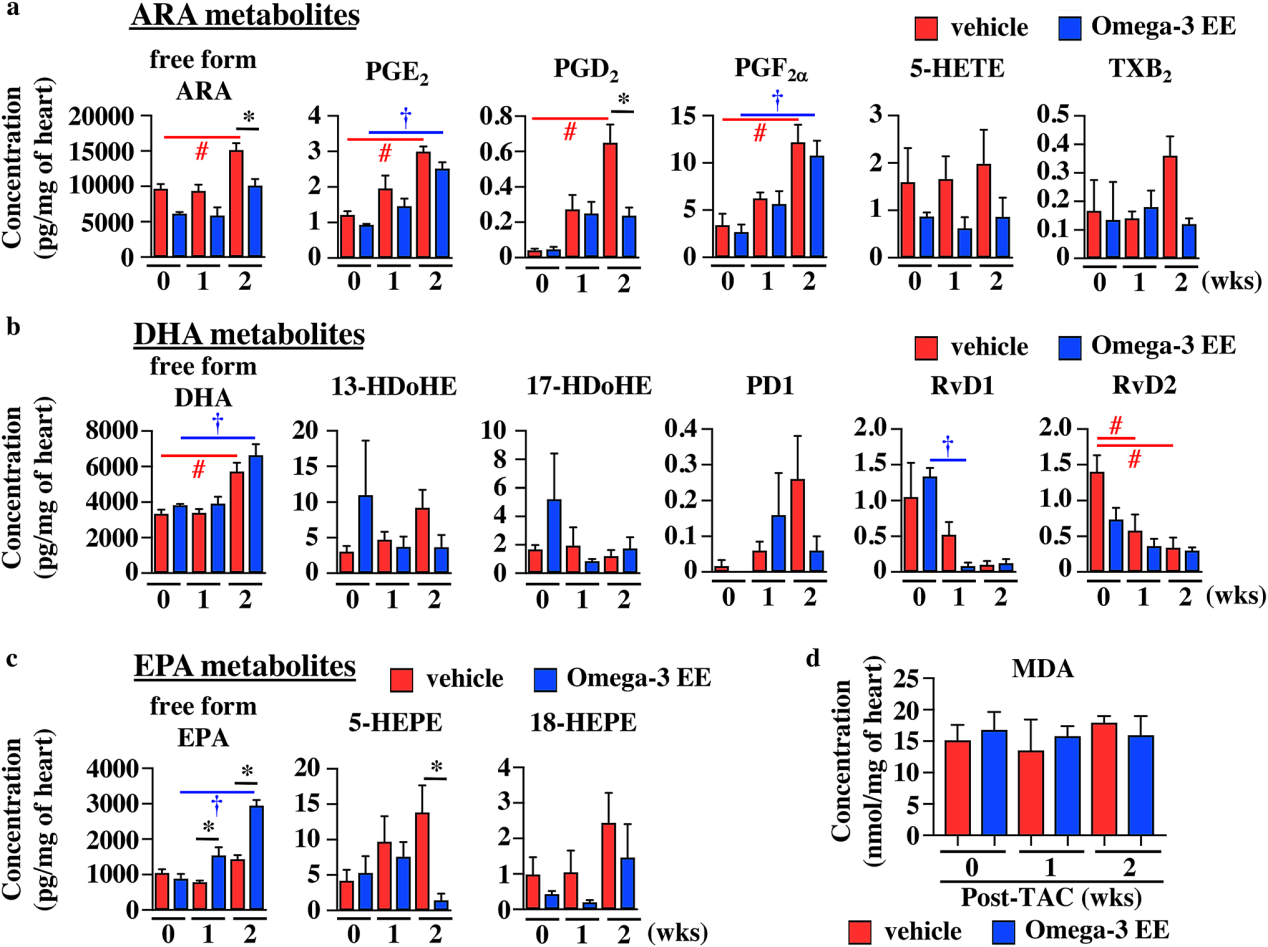
ARA-, DHA- and EPA-derived metabolites in the heart treated with Omega-3 EE. Arachidonic acid (ARA) (**a**), docosahexaenoic acid (DHA) (**b**), and eicosapentaenoic acid (EPA) (**c**) -related metabolites in heart were examined before (0), and 1 and 2 weeks (wks) after TAC surgery using liquid chromatography-tandem mass spectrometry. n = 3–6. *p < *0.05* compared between vehicle and Omega-3 EE groups. ^#^*p* < *0.05* compared with vehicle group before TAC*,* ^†^*p* < *0.05* compared with Omega-3 EE group before TAC. PG, Prostaglandin. HETE, Hydroxyeicosatetraenoic acid. TX, Thromboxane. HDoHE, Hydroxydocosahexaenoic acid. PD, Protectin D. Rv, Resolvin. HEPE, Hydroxyeicosapentaenoic acid. (**d**) MDA concentration in the heart was evaluated using TBARS assay. n = 4–7.

Metabolites of both omega-6 FA and omega-3 FA have been reported to be involved in regulating inflammation^13–17^. We thus evaluated the concentration of various metabolites during the development of cardiac hypertrophy by using liquid chromatography-tandem mass spectrometry (LC–MS) method^33,34^. The levels of free forms of ARA, EPA and DHA were much lower compared with those in total ARA, EPA and DHA, respectively (Figs. 3b and 4). Although total amounts of ARA and DHA in the heart were decreased after pressure overload (Fig. 3b), free forms of ARA and DHA were increased in the heart at 2 weeks after pressure overload in vehicle group (Fig. 4a,b). Some of ARA-related metabolites such as prostaglandin (PG) E_2_, PGD_2_ and PGF_2α_ were increased at 2 weeks after TAC operation (Fig. 4a).

We next examined whether oral treatment of omega-3 FA had an impact on concentration of free form and its metabolites of omega-3 FA and omega-6 FA in the heart. Under the condition without any pathological stresses, Omega-3 EE treatment did not show any effects on the concentration of free form of both omega-6 FA and omega-3 FAs (Fig. 4). Although free form ARA was increased in vehicle-treated group at 2 weeks after TAC operation, it was not increased in Omega-3 EE group (Fig. 4a). Free form DHA was increased under stress similarly both in vehicle and Omega-3 EE groups (Fig. 4b), while free form EPA was increased by pressure overload only in Omega-3 EE group (Fig. 4c).

There was no difference in any metabolites in the heart derived from omega-6 FAs and omega-3 FAs without pressure overload between vehicle and Omega3 EE groups (Fig. 4). Omega-3 EE treatment suppressed the concentrations of PGD_2_, an ARA-metabolite and 5-HEPE, an EPA-metabolite at 2 weeks (Fig. 4a,c), but the treatment did not have any effects on the concentration of DHA-derived metabolites (Fig. 4b).

Since ROS has been reported to be involved in heart failure^6^ and regulate the metabolites of omega-3 FA and omega-6 FA^21,35,36^, we examined whether ROS is involved in this process. We first evaluated malondialdehyde (MDA) concentration, a product of lipid peroxidation in the heart using the thiobarbituric acid reactive substance (TBARS) assay^37^. The concentration of MDA in the heart was not changed during the development of cardiac hypertrophy. Furthermore, there was no difference in the MDA concentration between vehicle and Omega-3 EE groups (Fig. 4d). We also evaluated iso-PGF2α, an isoprostane produced by the non-enzymatic peroxidation of ARA by LC–MS method^21,36^, but the level was below detection limit (Table 1). So, in this study we did not obtain any evidence suggesting that ROS modulates the metabolites of omega-3 FA and omega-6 FA in the development of cardiac hypertrophy.

**Table 1:** Undetectable metabolites.

	Undetectable metabolites
ARA metabolites	iso-PGF_2α_, 12-HETE, 15-HETE, LTB_4_, LXA_4_, ARA-*d*_*8*_, PGE_2_-*d*_*4*_, PGD_2_-*d*_*4*_, PGF_2a_-*d*_*4*_, 5-HETE-*d*_*8*_
DHA metabolites	7-HDoHE, 10-HDoHE, 14-HDoHE
EPA metabolites	12-HEPE, 15-HEPE, RvE1, RvE2, RvE3, PGD3, TXB3

*PG* prostaglandin, *HETE* hydroxyeicosatetraenoic acid, *LT* leukotriene, *LX* Lipoxin, *HDoHE* hydroxydocosahexaenoic acid, *HEPE* hydroxyeicosapentaenoic acids, *Rv* Resolvin.

### Omega-3 FA regulates inflammation in the development of cardiac hypertrophy

**Figure 5:**
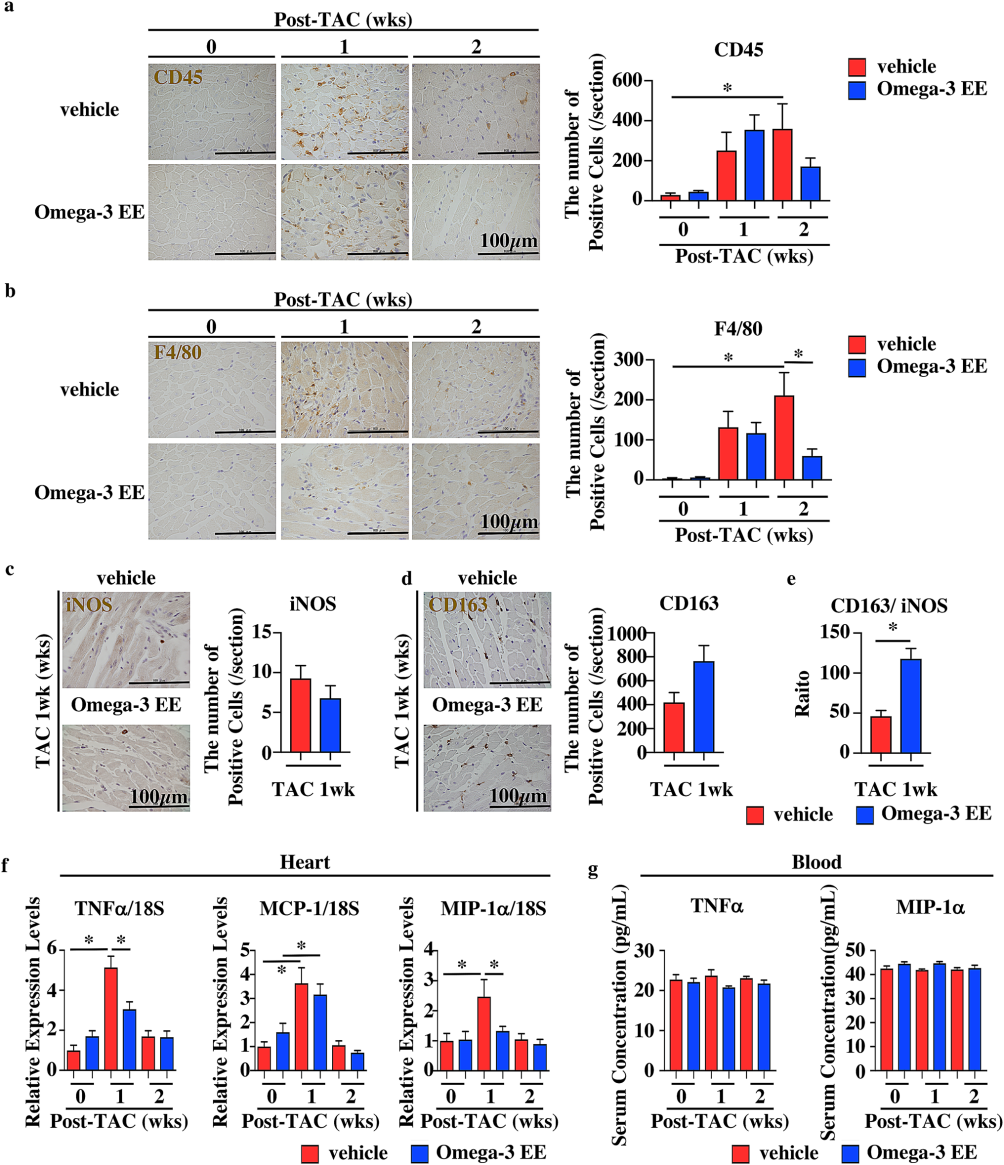
Omega-3 EE decreases the number of inflammatory cells with reduction of cytokine expressions in the heart. (**a**–**d**) Heart sections were stained with anti-CD45 (**a**), anti-F4/80 (**b**), anti-iNOS (**c**), and anti-CD163 (**d**) antibodies and the number of each antibody-positive cells was evaluated before (0) and after TAC surgery. * *p* < *0.05.* (**e**) The ratio of anti-CD163 positive cells to anti-iNOS positive cells was evaluated. * *p* < *0.05.* (**f**) Quantitative RT-PCR showed expression levels of TNFα, MCP-1 and MIP-1α in the heart. n = 5–10. *p < *0.05*. (**g**) Concentrations of TNFα and MIP-1α in blood were evaluated by ELISA. n = 4–7.

We finally examined whether oral administration of Omega-3 EE had some effects on inflammation in the heart. Many inflammatory cells such as CD45-positive leukocytes and F4/80-postive macrophages were still observed in hearts treated with vehicle at 2 weeks after operation, while the number of inflammatory cells went back to basal levels in the hearts treated with Omega-3 EE (Fig. 5a,b). Macrophages can be classified into several types; M1 macrophage is a proinflammatory phase macrophage and M2 is a resolution phase macrophage^38,39^. We examined the polarity of macrophages at 1 week after exposure to pressure overload when there was no difference in the number of F4/80-postive macrophages between groups (Fig. 5b). We used anti-iNOS and anti-CD163 antibodies to evaluate M1 and M2 macrophages, respectively^40^. There was no difference in the number of iNOS-positive cells and CD163-positive cells between vehicle and Omega-3 EE groups at 1 week after TAC (Fig. 5c,d), but the ratio of anti-CD163 positive cell numbers to anti-iNOS positive cell numbers was higher in Omega-3 EE treated group than that in vehicle group (Fig. 5e), indicating that omega-3 FA modulates the balance between M1 and M2 macrophages.

We also examined expression levels of proinflammatory cytokines in the heart and their concentrations in blood. Omega-3 EE attenuated the induction of inflammatory cytokines such as TNFα and MIP-1α expressions in the heart at 1 week (Fig. 5f). Both TNFα and MIP-1α concentrations in blood were not increased in 1 and 2 weeks after operation (Fig. 5g). These results suggest that the effects on the heart might be direct actions on the heart of omega-3 FA rather than indirect actions via systemic changes of metabolism.

## Discussion

Hearts use a lot of FAs and produce adenosine triphosphate (ATP) to sustain beating and ion exchange. Decreases of ATP have been reported to result in the development of heart failure^41^, but it remains unclear whether FAs were changed in the heart during the process of cardiac remodeling. This study showed that almost all FAs in the heart were decreased from 1 week after exposure to high blood pressure when cardiac systolic function was still preserved (Figs. 1 and 3b). There are three possibilities for the decrease of FAs in the heart; the decrease in influx of FAs into the heart, the increase in consumption, and the dilution of total FAs. Since it has been reported that FA uptake into the hypertrophied heart was inhibited by various factors such as the increase of ketone bodies and the decrease of FA transporters’ expressions^42,43^, the impairment of FA uptake might be a reason for the decrease of FAs in the heart. On the other hand, the second reason may be not the case, because there are reports indicating that the consumption of FAs is decreased in the pathological heart due to the impairment of mitochondrial function^7,44,45^. During the development of cardiac hypertrophy, protein synthesis is robustly enhanced and the cellular edema is also induced, the concentration of FAs might be relatively decreased.

There are some studies to examine the molecular mechanisms of how omega-3 FA preserves cardiac function under various stresses. Especially, Stanley WC and his colleagues demonstrated the role of omega-3 FA^7,45–47^. They showed that fish oil including much omega-3 FA increased the proportion of omega-3 FAs such as DHA and EPA and decreased that of ARA in mitochondrial phospholipids in the heart^7,45^ and suppressed the mitochondrial permeability transition pore opening^45^, which led to inhibition of cardiac dilatation under the pressure overload. We examined composition of total cardiac FAs at shorter time points after exposure to stresses. The change of total cardiac FA composition in our study (Fig. 3b) was almost similar to that in their studies, suggesting that Omega-3 EE also ameliorates cardiac dysfunction through the preservation of mitochondrial function.

Inflammation is a key factor in the development of cardiac hypertrophy and dysfunction induced by high blood pressure^7–9,48^. Inflammation has two processes, initiation and resolution, both of which are active reactions^39,49^. Omega-6 FA- and omega-3 FA-derived metabolites have been reported to be critically involved in both processes^13–17,50^, and the imbalance of these metabolites causes amplification and long-term continuation of inflammation, leading to tissue damage^49^. Accelerating resolution of inflammation contributes to maintaining organ homeostasis. Omega-3 FA’s metabolites have been reported to promote resolution^14–17^ and protect the heart from myocardial infarction and ischemia–reperfusion injury^51^. *Fat1* transgenic mice, which have an enzyme of *C. elegans* to convert omega-6 FAs to omega-3 FAs, preserved cardiac function under pressure overload via production of 18-HEPE, an EPA derivative^20^. These results suggest that promoting resolution by omega-3 FA metabolites has a therapeutic potential for cardiac diseases. In this study, we clearly showed that the oral administration of omega-3 FA prevented the development of heart failure by suppression of inflammation (Figs. 1 and 5).

In addition to the increase in free form EPA, it is an important point that Omega-3 EE had no effect on concentration of free form ARA and its metabolites at 1 week after imposing pressure overload (Fig. 4a). Until now, many drugs have been designed to inhibit the initial phase of inflammation when ARA-derived metabolites such as PGs and leukotrienes play crucial roles, and clinical trials and animal studies were performed to use these inhibitors to treat inflammation-related diseases. Recent clinical studies, however, revealed that a selective inhibitor of COX2, which is a key enzyme to convert a free form ARA to various types of lipid mediators, increased the risk of cardiovascular events^52,53^. Furthermore, deletion of PLA2, which is an enzyme to release omega-3 and omega-6 FAs from cellular membrane phospholipids, deteriorated ischemia–reperfusion injury in mice^54^. These results suggest that acute inflammatory responses are protective for hearts from various stresses.

There are some reports showing the role of PGD_2_ in both cardiomyocytes and macrophages in the ischemic heart. In cardiomyocytes, because of a low expression of PGD_2_ receptors (DP1 and DP2), PGD_2_ bound to PGF_2α_ receptor, and then showed a protective effect against ischemia–reperfusion injury via anti-oxidative effects^55^. In macrophages, the PGD_2_/ DP1 axis changed the polarity of macrophage to M2 macrophage, resulted in accelerated resolution of inflammation^56^. In this study, PGD_2_ concentration in the heart was higher in vehicle group than in Omega-3 EE group at 2 weeks after operation, while PGF_2α_ concentration was almost same between two groups (Fig. 4a), suggesting that PGD_2_ and PGF_2α_ might be not involved in cardiac remodeling in this study.

Regarding the omega-3 FA metabolites, Omega-3 EE decreased 5-HEPE concentration in the heart at 2 weeks after surgery (Fig. 4c). A report showed that 5-HEPE attenuated the expression of some proinflammatory cytokines induced by palmitic acid in macrophage^57^, but another showed that 5-HEPE did not reduce IL-6 production induced by macrophage-conditioned media in cardiac fibroblast^20^. We need further studies to examine the meaning of the reduction of 5-HEPE by Omega-3 EE during the process of cardiac hypertrophy.

Taken together, this study demonstrates that omega-3 resolves the inflammation at later stage but does not inhibit the initiation of inflammation and suggests that omega-3 might be an ideal drug to treat heart failure where inflammation is critically involved.

## Materials and methods

### Mouse experiments

All animal experiments were approved by the Ethics Committee for Animal Experiments of the University of Tokyo, and the procedures were adhered strictly to the guidelines for animal experiments of the University of Tokyo.

Ten-week old male C58BL/6J mice were purchased from CLEA Japan Inc. (Tokyo, Japan) and housed under controlled temperature with a 12 h light/ dark cycle and provided with standard food and water ad libitum. Mice were exposed to isoflurane and then were subjected to pressure overload by ligating aortic arch to induce cardiac hypertrophy^27^. Mice were treated with Omega-3 EE 1.5 mg/g of body weight^30^ via feeding needle every day from 5 days before operation to the end of study. Just before administration, a proper dose of Omega-3 EE was diluted to 100 µL with 0.5% methylcellulose to keep their stability. 0.5% methylcellulose was used as a vehicle. Omega-3 EE is a clinical drug containing both EPA and DHA, obtained from BASF AS (Norway) and Takeda Pharmaceutical Company (Japan)^29^. To check cardiac function and morphology, echocardiogram was performed without anesthesia using the Vevo2100 ultrasound system (FUJIFILM VisualSonics, Japan)^27^. We obtained hearts at 0, 1, 2 and 4 weeks after operation.

### Morphology

Hearts were fixed with 20% formalin (Sakura Finetek Japan Inc.) and embedded in paraffin. After cutting 4 µm each, paraffin sections were stained with anti-CD45 (BD Biosciences, USA), anti-F4/80 antibodies (Bio-Rad, USA), anti-iNOS antibody (abcam, UK), and anti-CD163 antibody (abcam) to evaluate cell infiltration. Elastica van Gieson staining and wheat germ agglutinin staining were performed to examine fibrosis proportion and calculate cell area, respectively. Cross-sectional area (CSA) of myocytes with a centrally located nucleus and overall circular shape was measured in the left ventricular free walls using Image J (NIH, USA)^27^.

### Fatty acid composition in plasma and heart

Samples for FA levels in the plasma and heart were prepared and analyzed by a modification of the one-step reaction analysis using gas chromatography^31,32^. For analysis of the FA composition in plasma and heart, 0.005% butylated hydroxytoluene /methanol and tricosanoic acid as internal standard, were added to samples and then kept at − 30 °C. The samples were heated at 98 °C for 1 h after addition of acetyl chloride. After the sequential addition of 0.5 M sodium hydroxide /10% sodium chloride and octane, samples were shaken for 3 min, centrifuged at 950×*g* for 10 min at 20 °C and the top layer was collected. The FA composition was measured using the Agilent 6850 A gas chromatograph (Agilent Technologies, USA) equipped with a flame ionization detector and an automatic sampler. Each FA methly esters were determined compared with retention time of standard materials. In the present study, we have determined FA levels in total FAs but not in the phospholipid^58^.

### Fatty Acid Metabolites in heart^33,34^

#### Sample preparation

Heart tissues were homogenized with 70% methanol and centrifuged at 5000×*g* for 10 min at 4 °C. The supernatants were diluted with distilled water. PGE_2_-*d*4, PGD_2_-*d*4, PGF_2α_-*d*4, 5-HETE-*d*8, and ARA-*d*8 were added to each sample as internal standards. After adjusting the PH of samples to 4.0 using 0.1 M HCl, samples were applied to preconditioned solid-phase extraction cartridges (Sep-Pak C18, Waters, USA). Sep-Pak cartridges were washed with water and n-hexane. Finally, methyl formate was applied to elute FA metabolites. The precise method was described as previously^33,34^.

#### LC–ESI–MS–MS-Based Analysis

High-performance liquid chromatography (HPLC) was combined with electrospray ionization- mass spectrometry (ESI–MS). HPLC was performed using a Luna 3u C18(2) 100 Å LC column (100 × 2.0 mm, Phenomenex, USA). Samples were eluted in a mobile phase comprising acetonitrile–methanol and water–acetic acid in a 27:73 ratio for 5 min, ramped up to a 100:0 ratio after 25 min, and held for 10 min. Tandem mass spectrometry (MS–MS) analyses were conducted in negative ion mode. FA metabolites were detected and quantified by selected reaction monitoring (SRM as listed in Table 2). Each area was calculated using the Xcalibur 2.1 software (Thermo Fisher Scientific, USA). The precise method was described as previously^33,34^.

**Table 2:** SRM transitions of fatty acid metabolites.

ARA metabolites		DHA metabolites		EPA metabolites	
Compound	SRM transition (m/z)	Compound	SRM transition (m/z)	Compound	SRM transition (m/z)
ARA	303 > 259	DHA	327 > 283	EPA	301 > 257
PGE_2_	351 > 271	7-HDoHE	343 > 141	5-HEPE	317 > 115
PGD_2_	351 > 271	10-HDoHE	343 > 153	12-HEPE	317 > 179
PGF_2α_	353 > 193	13-HDoHE	343 > 193	15-HEPE	317 > 219
Iso-PGF_2α_	353 > 193	14-HDoHE	343 > 205	18-HEPE	317 > 259
5-HETE	319 > 115	17-HDoHE	343 > 245	RvE1	349 > 195
12-HETE	319 > 179	PD1	359 > 153	RvE2	333 > 115
15-HETE	319 > 219	RvD1	375 > 141	RvE3	333 > 213
TXB_2_	369 > 169	RvD2	375 > 175	PGD3	349 > 233
LTB_4_	335 > 195			TXB3	367 > 169
LXA_4_	351 > 115				
ARA-*d*_8_	311 > 267				
PGE_2_-*d*_4_	355 > 275				
PGD_2_-*d*_4_	355 > 275				
PGF_2α_-*d*_4_	357 > 197				
5-HETE-*d*_8_	327 > 116				

*PG* prostaglandin, *HETE* hydroxyeicosatetraenoic acid, *TX* Thromboxane, *LT* leukotriene, *LX* Lipoxin, *HDoHE* hydroxydocosahexaenoic acid, *PD* Protectin D, *Rv* Resolvin, *HEPE* hydroxyeicosapentaenoic acids.

### Quantitative RT-PCR (qRT-PCR)

RNA was purified from heart samples using Trizol solution (Thermo Fisher Scientific) according to a manufactural protocol. Expression levels of TNFα, MCP-1, and MIP-1α in the heart were evaluated by qRT-PCR method. Primer designs were decided in reference to PrimerBank^59^. Data were normalized to 18S ribosomal RNA. A precise method was described as previously^27^.

### Cytokine and chemokine concentration in blood

ELISA was performed according to the manufacturer's instructions to examine the levels of TNFα and MIP-1α (R&D Systems, USA) in blood.

### TBARS assay

Lipid peroxidation level in the heart was measured using TBARS assay kit (Cayman Chemical Company, USA), as described previously^37^, and data were expressed as moles of MDA/mg protein of heart.

### Statistical analysis

Data are shown as mean ± SEM. Statistical analysis was performed using GraphPad Prism (GraphPad Software, USA). Multiple group comparison was performed by one-way analysis of variance (ANOVA) or two-way ANOVA followed by the Bonferroni procedure for comparison. Values of *P* < 0.05 were considered statistically significant.
